# Development of a conceptual model for research on cyclical variation of patient reported outcome measurements (PROMs) in patients with chronic conditions: a scoping review

**DOI:** 10.1186/s41687-021-00395-x

**Published:** 2021-11-04

**Authors:** A. F. Davey, J. Coombes, I. Porter, C. Green, A. J. Mewse, J. M. Valderas

**Affiliations:** 1 Health Services and Policy Research Group, Exeter Collaboration for Academic Primary Care, NIHR PenARC, University of Exeter Medical School, University of Exeter, Exeter, UK; 2 Health Economics Group, Institute of Health Research, University of Exeter Medical School, University of Exeter, Exeter, UK; 3 Exeter Collaboration for Academic Primary Care (APEx), University of Exeter Medical School, University of Exeter, Exeter, UK; 4 Present Address: Department of Psychology, College of Life and Environmental Sciences, University of Exeter, Exeter, UK

**Keywords:** Patient reported outcome measurement, Scoping review, Variability, Time-dependent

## Abstract

**Background:**

Although circadian, seasonal, and other cycles have been observed for a number of chronic conditions, their impact on patient reported outcomes measurements (PROMs) has not been systematically explored, rendering our understanding of the effect of time of measurement on PROM scores very limited. The aim was to conduct a scoping review to determine what is known about how intra-individual cyclical variation might affect the way individuals with chronic conditions respond to patient-reported outcome measures.

**Methods:**

A protocol of a systematic scoping review was registered on PROSPERO (CRD42017058365). We developed a search strategy based on previous relevant reviews and implemented it in: MEDLINE, Embase, PsycINFO, and CINAHL. No restrictions were placed on article types and backward and forward citation searches were conducted. Screening and data extraction were independently completed by up to four reviewers. An adapted version of CASP criteria was used to appraise the quality of included articles. Concepts that were important in understanding the impact of cyclical variation on PROM scores were elicited from the papers and iteratively refined through discussion amongst the authors.

**Results:**

2420 references resulted from the searches, with 33 articles meeting the inclusion criteria. Most study designs included observational research (particularly ecological momentary assessment), 2 were RCTs and 2 were systematic reviews. Studies mainly focused on specific health conditions: mental health, respiratory and musculoskeletal. There was a lack of qualitative research and theoretical framework to explore these concepts more fully. Five overarching concepts emerged: variation in outcomes, variation of scores, psychological status, individual factors, and environmental/situational factors. A conceptual model was developed outlining the relationships between these concepts.

**Conclusions:**

There is empirical evidence that supports cyclical variation in PROM scores across different chronic conditions, with potential very significant implications for administration and interpretation of PROMs. The proposed conceptual model can support further research in this area.

**Supplementary Information:**

The online version contains supplementary material available at 10.1186/s41687-021-00395-x.

## Background

Cyclical variation of physiological and clinical variables has been observed in relation to biological rhythms for different periods, including both circadian (24-h period) and longer infradian periods (e.g. circaseptan (week), circamensual (month), circannual (year)). Physiological cyclical variation has long been established for bodily temperature, blood pressure, fertility, weight, mood and sleep [1, 2]. Diseases demonstrate cyclical variation in relation to physiological changes that occur, with risks increased for mortality at different hours of a 24-h period for certain conditions [3]. The risk of a cardiac event (e.g. myocardial infarction, ST-segment depression), for example, is much greater in the earlier hours of the morning due to the surge of blood pressure at waking, whilst peak expiratory flow and forced expiratory volume in people with asthma are greater during the daytime and poorest at night, and cortisol exhibits high-amplitude circadian rhythmicity highlighting the importance of when blood samples should be tested [3].

Cyclical variation can be also anticipated to impact on patient assessments of their health. Patient reported outcomes (PROs) are health outcomes, which are directly reported by an individual without an interpretation of the response by a clinician or anybody else [4]. PROs include the symptoms people experience, their functioning (functional status), general perceptions of their health, health related quality of life and well-being [5, 6]. PROs are complementary to objective outcomes that are frequently used in clinical settings (e.g. blood pressure, temperature, blood measurements) and are collected using instruments known as patient reported outcome measurements (PROMs). They provide unique and essential information on patients’ perceptions of both the impact of conditions and their management, information that is essential for patient centred decision making [7, 8]. The process by which an individual assesses their health is a complex interaction between determinants of health, the time of measurement, and the constructs measured by the instrument. Better understanding cyclical variations in the outcomes of health conditions can support patients in managing their condition. Knowing when their conditions are at its best and worst can help patients manage their daily lives improving their quality of life. Clinicians generally focus on persistent symptoms at the time of patient consultations, and overlook fluctuations that occur throughout the day, week or month. Fluctuating patient reported outcomes over time reported by patients could have a bearing on clinician’s evaluation of treatment plans and understanding of their patient’s disease progression. If there is variation then both patients and clinicians can tailor management of the health condition.

Intra-individual cyclical variation has previously received little attention in the field of PROMs. PROMs were originally conceived for obtaining valid and reliable estimates of outcomes at a group level for measuring disease burden or evaluating health care interventions for populations [9–11]. In this context, intra-individual cyclical variation may have become less observable as it would be eventually diluted when multiple individual scores are aggregated for obtaining group estimates, particularly when cyclical variation is expected to be distributed at random across different groups of patients in randomised clinical trials. However, the increased use of these measurements in clinical practice, for example in psychological services such as IAPT (Improving Access to Psychological Treatment) [12] for individual patients makes unaccounted intra-individual cyclical variation essential for establishing whether a difference in scores in a patient signals a true change in patient health status, rather than being due to expected cyclical variation.

The impact of cyclical variation on patient reported outcomes measurements has not been systematically explored. This renders our understanding of the effect of time of measurement on PROM scores very limited. The aim was to determine how intra-individual cyclical variation has been previously defined and measured and how it might affect the way individuals with chronic conditions respond to patient-reported outcome measures. Development of a conceptual model can provide hypotheses to be tested in the management of the variation that occurs.

## Methods

We conducted a scoping review, [8], as this is the method of choice for the proposed aims [13, 14].

### Search strategy and selection of the literature

A search was conducted for relevant articles in four databases: MEDLINE (In-Process & Other Non-Indexed Citations and Ovid MEDLINE, 1948 to Present, accessed through OvidSP); Embase (1974 to present, accessed through OvidSP); PsycINFO (1967 to present, accessed through OvidSP); and CINAHL (from 1981 to present, accessed through EBSCO) using a model pre-defined strategy developed through an iterative process, based on published searches and with input from an information specialist. The searches were conducted between July and September 2017 and there were no restrictions to the time period being searched. The search strategy was developed and reviewed with an information specialist from the systematic reviewing team. The strategy was tested prior to implementation in order to assess the types of papers resulting from the search. The final strategy with search terms were organised into four blocks: PROs, measurement, time, and chronic conditions. For example, within the PROs block search terms included symptom, function, quality of life. The full search strategy across the different databases is included as a Additional file 1: Table 1. The search strategy was adapted to each database to comply with their terminology [15].

**Table 1:** Characteristics of the articles included in the review

References	Country	Design	Setting	Conditions	PROMs measurement		
					Frequency^a^	Data collection period^b^	Study quality^c^
aan het Rot et al. 2012	Netherlands	Systematic Review	Various settings	Depression	Between 3 and 10	Varied	3
Abdel-Kader et al. 2014	USA	Observational (Longitudinal)	Secondary care	Kidney disease	4	7 d	3
Bellamy et al. 1991	Canada	RCT^d^	Secondary care	Rheumatoid arthritis	6	9 d	1
Bromberg et al. 2014	USA	Observational (Longitudinal)	Secondary care	Arthritis	3	1 m	3
Claros-Salinas et al. 2010	Germany	Observational (Longitudinal)	Secondary care	Multiple sclerosis	3	2 d	2
Crosby et al. 2009	USA	Observational (Longitudinal)	Community	Eating disorders	6	2 w	3
Curran et al. 2004	USA	Observational (Longitudinal)	Secondary care	Cancer	4	5 d	3
Dekkers et al. 2000	Netherlands	Observational (Longitudinal)	Community	Rheumatoid arthritis	8 (PROMs) 9 (saliva)	2 d	2
de Wit et al. 1999	Netherlands	RCT	Secondary care	Cancer	2	2 m	6
Feuerecker et al. 2015	Germany	Observational (Longitudinal)	Secondary care	Chronic dizziness	5	1 d	3
Feys et al. 2012	USA, Spain, Belgium, Finland, Denmark	Observational (Longitudinal)	Secondary care	Multiple sclerosis	3	1 d	3
Graham-Engeland et al. 2016	USA	Observational (Longitudinal)	Community	Rheumatoid arthritis	5	7 d	2
Hamilton et al. 2007	USA	Observational (Longitudinal)	Secondary care	Fibromyalgia	7	2 d	2
Hardt et al. 1999	Germany	Observational (Cross-sectional)	Secondary care	Chronic pain	1	1 d	5
Houtveen et al. 2015	Netherlands	Observational (Longitudinal)	Community	Mental disorder	4	3 w	3
Kikuchi et al. 2012	Japan	Observational (Longitudinal)	Secondary care	Headache	4	7 d	3
Kleiman et al. 2017	USA	Observational (Longitudinal)	Community	Mood disorders	4	28 d	2
Kratz et al. 2016	USA	Observational (Longitudinal)	Community	Chronic pain	5	7 d	5
Lavender et al. 2013	USA	Observational (Longitudinal)	Secondary care	Eating Disorders	6	2 w	4
McCarley et al. 2007	USA	Observational (Longitudinal)	Community	Chronic Obstructive Pulmonary Disorder (COPD)	5	8 d	7
Okifuji et al. 2011	USA	Observational (Longitudinal)	Secondary care	Fibromyalgia	3	30 d	2
Partridge et al. 2009	Europe and USA	Observational (Cross-sectional)	Community	Chronic Obstructive Pulmonary Disorder (COPD)	1	1 day	2
Pfaltz et al. 2010	Switzerland	Observational (Longitudinal)	Community	Mood disorders	5	8 d	3
Powell et al. 2017	UK	Observational (Longitudinal)	Secondary care	Multiple sclerosis	6	4 d	7
Roche et al. 2013	France	Systematic Review	Various settings	Chronic Obstructive Pulmonary Disorder (COPD)	Varied	Varied	3
Schanberg et al. 2005	USA	Observational (Longitudinal)	Secondary care	Polyarthritic arthritis	1	2 m	6
Schlager et al. 1995	USA	Observational (Cross-sectional)	Primary care	Depression	1	1	5
Schwartz 2000	USA	Observational (Longitudinal)	Community	Cancer	1	8 w	4
Sewell et al. 2010	UK	RCT	Secondary care	Chronic Obstructive Pulmonary Disorder (COPD)	Twice (pre and post)	7 w (pre and post)	4
Shin and Lee 2014	Korea	Observational (Longitudinal)	Secondary care	Chronic pelvic pain	Every 2–3 months	27 m	3
Stinson 2008	Canada	Observational (Longitudinal)	Secondary care	Arthritis	3	2 w	2
Tsanas et al. 2016	UK	Observational (Longitudinal)	Secondary care and community	Depression	1	3 m	2
Vernon et al. 2010	USA	Observational (Longitudinal)	Primary care	Chronic cough	1	2 w	3

^a^The number of times measurements were completed in a day

^b^d: days; w: weeks; m: months

^c^A lower score on the CASP means article focused less on cyclical variation of PROMs (range from 0 to 7)

^d^Randomised Controlled trial

Due to the nature of the type of literature review the inclusion criteria to screen the titles, abstracts and full-text articles that was set included any record (e.g. original studies, systematic reviews, editorials, conference proceedings etc.) that met all of the following criteria:

1. Reporting PROMs data (ranging from multiple scales to single items)
2. Reporting variation of PROMs across time (e.g. daily, weekly, monthly, seasonally, etc.)
3. Including patients with one (or more) chronic condition(s)
4. Having been written in English

The inclusion criteria were tested by four reviewers (AD, JC, IP and JMV) firstly on a selection of 20 titles and abstracts and then on full-texts and inter-rater reliability between the each pair of reviewers was calculated through the Cohen’s Kappa statistic [16] in Excel. The median kappa across all reviewers at abstract stage was 0.92, whilst at full-text stage the median was 0.96. All the titles and abstracts were imported and reviewed using Rayyan, a web-based platform to facilitate the screening process [17]. When reviewers agreed that abstracts did not meet the eligibility criteria these were excluded. Full-text screening took place following this process. Full-text articles that were not available through the University library system were obtained by emailing authors or contacting them via ResearchGate. A final list of full text articles was compiled and backwards and forwards citation searching on the included full text articles was manually undertaken [18]. Backward citation searches were conducted by examining each source cited by the references in each article. Forward citation searches were conducted by identifying articles that cited the included articles in PubMed.

### Data extraction and quality assessment

Information was extracted into an Excel spreadsheet using a pro-forma including the characteristics of the articles, data collection methods and time periods, explicit (existing conceptual models, explicit hypotheses on cyclical variation of PROMs) and implicit assumptions (associations being explored without a priori hypotheses). In order to assist in categorisation of the chronic conditions, the World Health Organisation’s International Statistical Classification of Diseases and Related Health Problems guide (ICD-11) was used [19].

The quality of included articles was assessed using an adapted version of the Critical Appraisals Skills Programme (CASP) tool of observational research to assess whether articles focused on time-related variation in their studies [20] (Additional file 2: Table 2). The CASP tool is frequently used by systematic reviewers to assess the quality of articles and there are tools designed for a variety of study designs (e.g. qualitative, randomised controlled trials, and observational). Quality assessment methods were tested in a pilot evaluation prior to use across the literature by AD, CG and JMV.

### Evidence synthesis

**Fig. 1:**
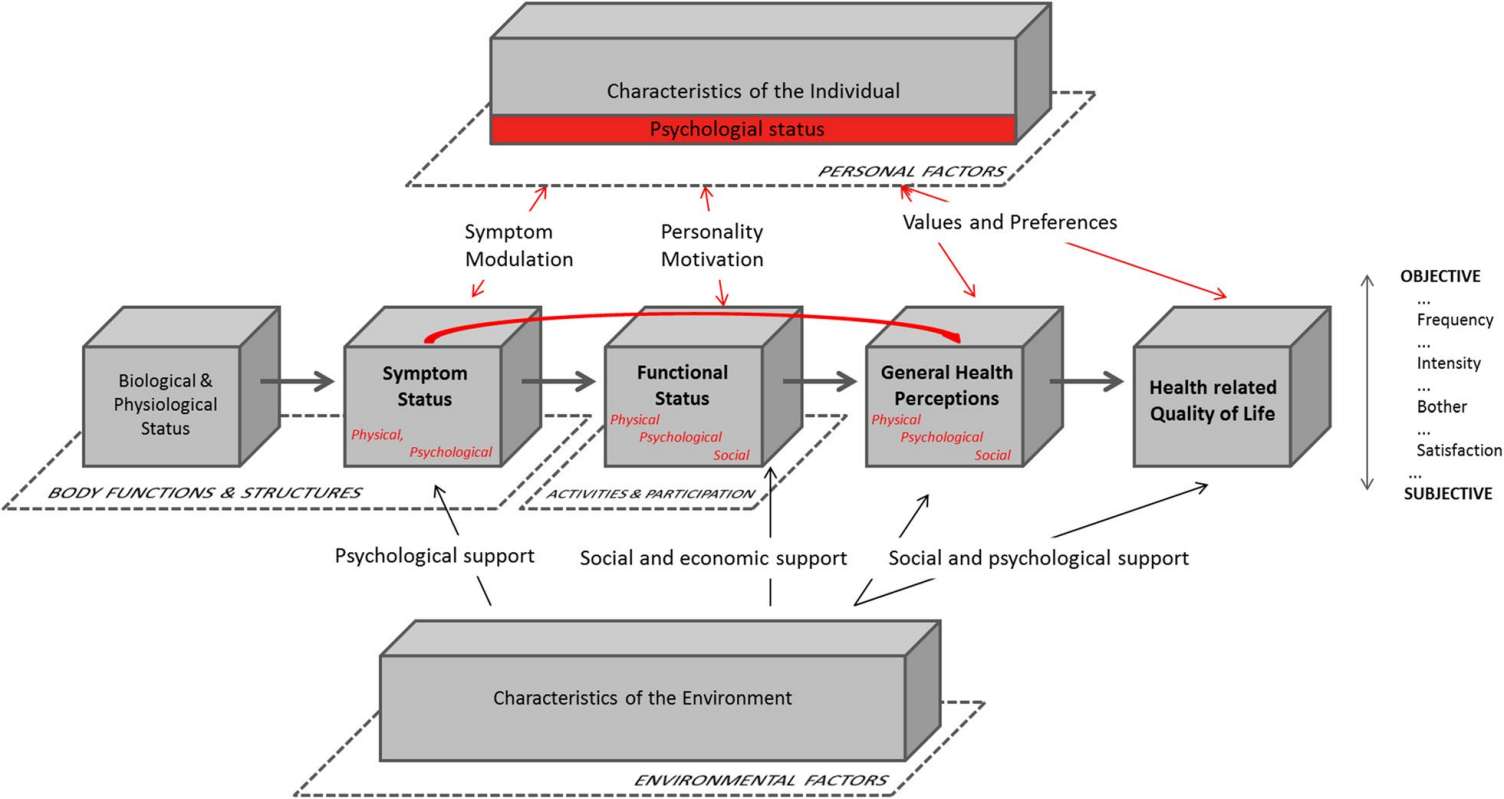
Revised Valderas and Alonso model of Patient Reported Outcomes [19]

The conceptual model considered what needed to be taken into account and the models (Valderas and Alonso’s classification model, Fig. 1 [5, 21]) helped to inform this. The developed conceptual model further elaborated on the variables and hypothesised relationships in the explicit and implicit assumptions of the studies included in the review. In particular, the studies and conceptual model uniquely highlighted how psychological status is both a functional outcome as well as a determinant of patient reported outcomes. AD and JMV conducted a pilot for the approach, AD subsequently extracted key concepts explaining time-related variation of scores from all studies. This was done by going extracting the key relevant concepts and their proposed associations as mentioned in each document (e.g. health outcomes, biorhythms, etc.). These concepts and associations were then mapped out by AD, onto a conceptual model, which was iteratively refined through discussion amongst the authors.

## Results

**Fig. 2:**
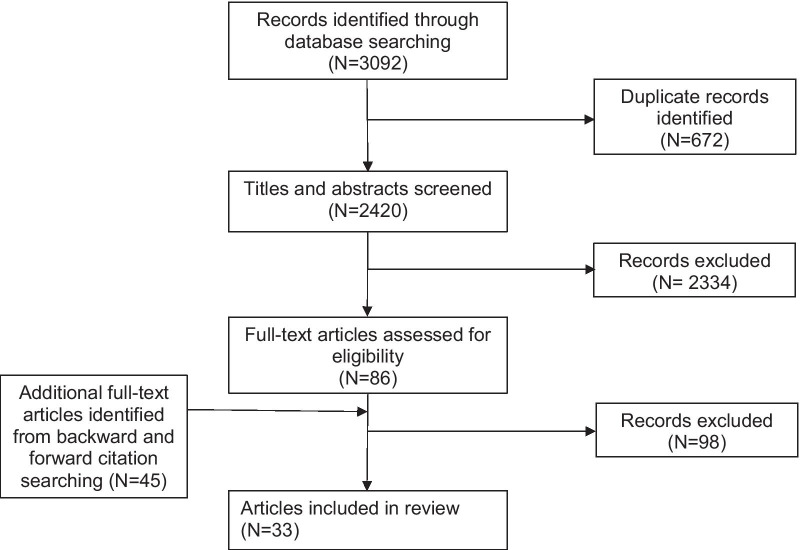
Flow chart of the scoping review

A total of 2420 articles were retrieved from bibliographic databases and an additional 45 full-text articles were identified through forward and backward citation searches (see Fig. 2). Inter-rater reliability between the reviewers were κ = 0.83 (abstract) and κ = 0.92 (full text). A total of 33 studies were included in the final review (Table 1). Articles that were excluded from the final review did not meet the inclusion criteria as outlined in the methods section, for example they did not study variation across time, or report PROMs scores. The quality of the studies varied from 3 to 7 points (maximum) on the adapted CASP tool, with three articles achieving the maximum score of 7.

### Study characteristics

The majority of the literature was published from 2000 [22–42], with ten articles published in the last five years [22, 30, 34, 35, 38, 40–45]. Seventeen studies were conducted in North America [22, 25, 27, 29, 30, 33–37, 42, 45–49], twelve in Europe [23, 24, 28, 31, 32, 38, 40, 41, 44, 50–52], with two studies across both regions [26, 29] and two further studies from Asia [43, 53]. Studies were conducted in patients with five broad disease categories: mental health (n = 8) [28, 31, 35, 40–42, 46, 48], musculoskeletal (n = 7) [23, 34, 36, 39, 45, 47, 54], respiratory (n = 5) [26, 27, 32, 33, 51], nervous system (n = 4) [29, 36, 37, 44], and other conditions (n = 8) [22, 25, 30, 38, 43, 49, 50, 53]. Studies sampled mostly adult populations (n = 30), with two studies focusing solely on female adults [36, 49], and the remaining focusing on children [34, 39, 47]. Studies recruited participants in specialist outpatient departments within secondary care (n = 18) [22, 24, 25, 29, 32, 34, 36–39, 43, 44, 47, 50, 52–55], primary care and the community (n = 11) [23, 26, 27, 30, 31, 40–42, 45, 46, 49]. The two systematic reviews did not have any inclusion criteria relevant to specific settings.

### Study designs

Included studies in the literature collected PROMs primarily for the measurement of symptom severity (such as pain, fatigue, stiffness, shortness of breath and affect (emotions)) and functional status, including disability measures. There was a lack of quality of life measures used across the articles. Many of the studies used visual analogue scales (VAS) for pain, fatigue and stiffness [24, 27, 29, 34, 39, 40, 47, 54]. Seven studies used single items on mood, pain and fatigue [23, 30, 36, 38, 39, 44, 45].

The included studies employed a range of only quantitative methodologies and designs, including observational (cross-sectional and cohort) [22–27, 29–31, 33, 34, 36–48, 50, 53, 55] and experimental (randomised controlled trials) designs [32, 52, 54]. We did not identify any studies using qualitative or mixed methods, commentaries, or editorials. Many of the studies using observational methods used the Ecological Momentary Assessment (EMA) approach to data collection [22, 23, 25, 30, 31, 36, 37, 40–42, 44–46, 53, 56, 57]. There were two systematic reviews which focused on methodological approaches to collecting real-time data in two specific conditions, depression and COPD [28, 51]. The majority of studies used a repeated measures design, collecting data from twice to eight times a day[22, 24, 25, 27, 29–31, 34, 36–39, 41, 42, 44, 46, 52–56], with one study collecting data every three to four months over a 27 month period [43]. Three studies were of cross-sectional design collecting data only once [26, 48, 50].

### Conceptual model

**Fig. 3:**
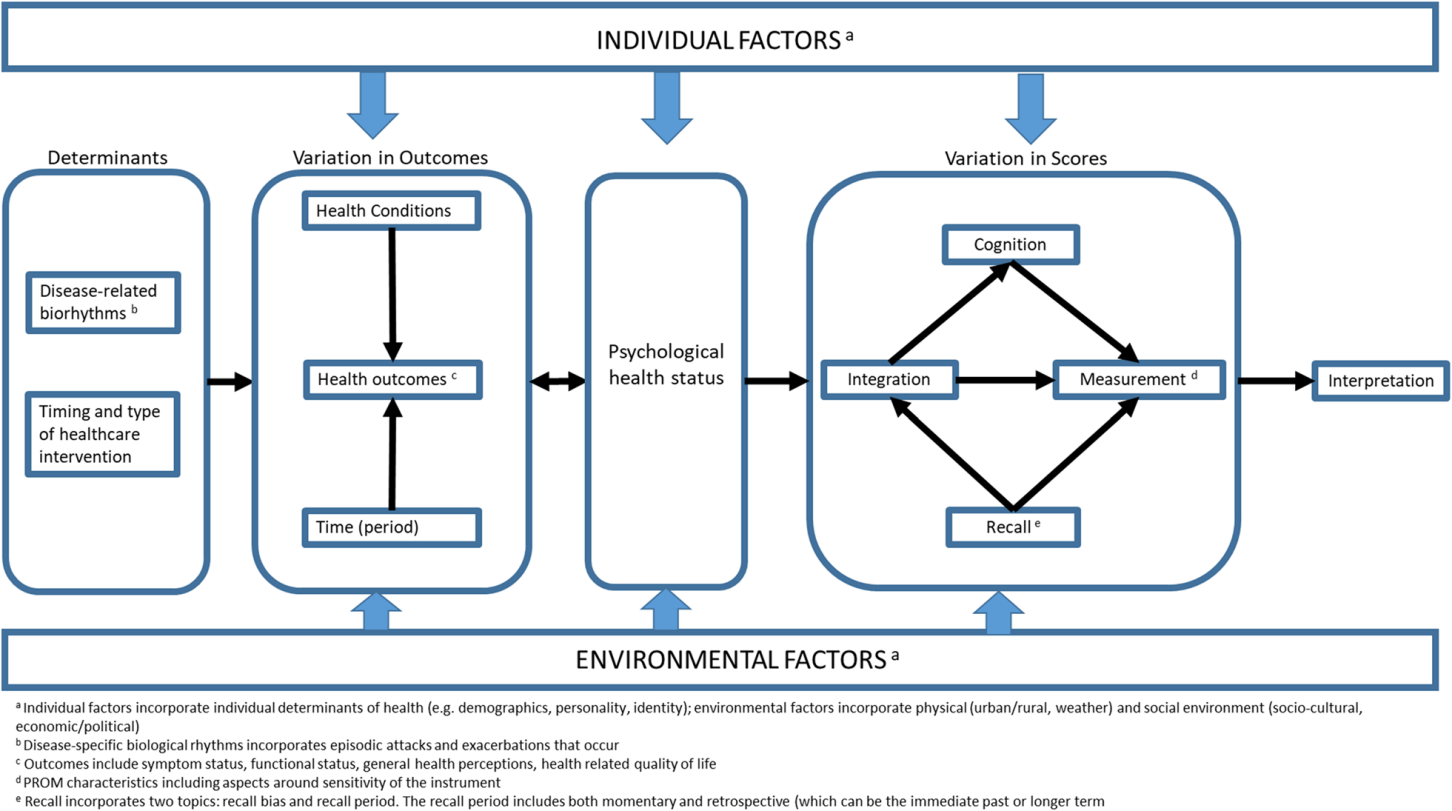
Conceptual model for cyclical variation of Patient Reported Outcome Measures (PROMs)

Two core constructs were identified in our conceptual model from the literature: variation in health outcomes (PROs), and variation in scores (PROMs) (Fig. 3). In addition the model considers two determinants (disease-related biorhythms, and timing of biomedical interventions), one key mediator (psychological status), and two main moderators (individual and environmental factors). The determinants only directly influence variation of outcomes, while the moderators impact on all of the two determinants, two core constructs, and the mediator. Psychological health status has a bidirectional relationship with variation in outcomes (an individual’s overall health has an impact on psychological state, which also influences overall health). All these interactions result in possible sources of variation in scores, and determine how scores are to be interpreted.

### Moderators: individual and environmental factors

One of the fundamental determinants of health is the person’s individual characteristics and behaviour. When considering individual factors, part of this can be defined in terms of the demographics (e.g. age, gender) of the population being studied, their personality, motivation, values and preferences. The impact of the concepts of motivation and personality are reinforced with research conducted by Hardt et al. [50] or Graham-Engeland et al. [45], linking personality characteristics such as mood-like traits to the experience of pain. An individual’s level of acceptance or determination changes the way they perceive their outcomes (e.g. symptoms, functional status), for example pain acceptance was seen to buffer expected increases in pain interference and decreases in physical activity in the context of high pain for spinal cord injured patients [30]. Individual thresholds could also determine changes in scores longitudinally, especially in relation to subtle changes in pain that occur for those with high pain thresholds. Multimorbidity adds to the complexity of completion and interpretation of PROMs and was an important concept to consider in the articles. Co-morbid conditions sharing similar symptoms can impact on how patients report on one particular condition, with symptoms in one condition (e.g. pain in rheumatoid arthritis) potentially triggering another condition (e.g. depression) [23, 45].

Environmental determinants of health include both the physical and social environment in which individuals live and work. The physical environment includes the natural setting (e.g. weather, bioenvironmental markers, etc.) and the human setting (urban/rural). For example, temperature changes over the year can impact on symptom status for COPD sufferers exacerbating their symptoms in the winter [32], limiting their participation in activities. Furthermore, cold weather has been associated with a breakthrough of chronic prostatitis/chronic pelvic pain syndrome symptoms in the winter compared to acute symptoms reported in the summer [43]. External rhythms, such as exposure to sunlight or external stimuli, have been linked to variation in outcomes and psychological status with increased sunlight linked to better outcome scores [28, 48], and worsening outcomes for long exposure to external stimuli [38]. Sleep quality was highlighted as a contributing factor to worsening PRO scores due to sleep disruption, triggered by numerous variables such as stress [34, 37, 49] or night-time symptoms [51] and effects on symptoms such as mood upon awakening, fatigue [23, 44, 49], and poor overall functioning [37].

### Determinants

Two main sources of outcomes variation are identified: disease-related biorhythms, and timing of health care interventions (including medication). Disease-related biorhythms are the natural cycles of change in the body’s chemistry or function and symptoms [26], related to the health condition, which function in a rhythmic pattern. For example, those with rheumatoid arthritis present a diurnal patterning with regard to their symptoms [23, 54], whilst cortisol levels that affect mood in seasonal affective disorder has a circannual rhythm [48]. These biorhythms govern certain health outcomes such as symptoms and function [26], and ultimately affect health related quality of life.

The timing of medical interventions (such as the dosage and pharmacokinetics of medication) is an important factor to consider as it has significant consequences on the variation in health outcomes, due to both their indications and adverse effects [22, 26, 33, 53]. Cancer treatments have severe effects on individuals’ symptoms and functional ability. Breast cancer patients present a distinct infradian patterning of fatigue levels following chemotherapy treatments, typically highest within 24 to 48 h following treatment [49]. The type of intervention prescribed (whether that be pharmacological or not) for every condition will be different and will have varying levels of impact on an individual’s overall outcome. In some conditions, the time of year an intervention is administered, such as rehabilitation, impacts on overall health outcomes post-completion. For example, Sewell et al. [32] showed that for COPD patients seasonal variations have an important impact on functional performance after pulmonary rehabilitation.

### Variation in health outcomes

Variation in health outcomes depends on health conditions, the type of health outcomes (as outlined in the existing models/classification systems on health outcomes), and time (periods). The studied health conditions show cyclical patterns in their effects on health outcomes such as symptom and functional status, and health related quality of life. Individuals with musculoskeletal and nervous system conditions experience a diurnal patterning of symptoms during the day, with fatigue and pain worsening by the end of the day [23, 24, 29, 34, 36, 37, 39, 41, 44, 45, 47, 53, 54]. However, individuals with respiratory conditions experience a different diurnal patterning of symptoms whereby symptoms are worse in the morning and evening [26, 27, 33, 51]. In addition, respiratory conditions have seasonal patterning with individuals reporting increased symptom severity levels over winter months [32].

Functional status, one’s ability to perform daily tasks, varies with health conditions and time [30, 32, 34, 39, 41, 48]. It is apparent that one’s functional status presents a diurnal and infradian rhythmic patterning depending on the health condition. For example, functional performance for COPD patients worsens in the winter months [32], greater functional difficulties are experienced in the mornings and on the days following nights of poorer perceived sleep quality for arthritis sufferers [34, 39].

Although health related quality of life (HRQoL) was not extensively researched in the papers, there was some acknowledgement of the association between HRQoL and the symptoms and functioning experienced by individuals [24–27, 29, 30, 39, 40, 43, 51, 56] with regard to fluctuations in symptoms and functioning across conditions being associated with lower health related quality of life. It is evident that fluctuating health outcomes has a bi-directional relationship with an individual’s psychological status, in that mood is affected by and affects symptoms, functioning and health-related quality of life.

### Mediator: psychological health status

Although psychological health status is also a health outcome, it has been presented as a mediator in this model. The rationale behind this is that psychological health status strongly impacts on and is impacted by all the other concepts in the model. The mental state an individual is in appears to be determined by the two moderators as well as the other health outcomes. The other concepts within the model influence the (non-observable) mediator concept (psychological health), which in turn influences variation in scores. Psychological health status incorporates mood (e.g. emotions), cognition and general psychological and mental functions. An individual’s psychological health status is determined by both the individual and environmental variables. In our model psychological health status is a mediator between variation of PROs and variation in the scores. A change in psychological status resulting in worse outcome scores has been observed for patients with MS [44], arthritis [47], or suffering mental health problems. Variations in mood have been linked to fluctuations in pain, stiffness, and fatigue in children with chronic arthritis [34]. As represented in the model, the relationship between psychological status and variation of outcomes is bidirectional. Bulimic patients, for example, tended to engage more in bulimic behaviour on days where negative emotion is high, and vice versa. In addition, mood measured in a previous month predicted pain severity in the next month [45].

Psychological health status also played a role in the prediction of reduced social activities for children with chronic arthritis demonstrating the link it has with functional status [47], with lower mood and stiffness being a predictor of school attendance. The relationship between psychological status and variation of scores is unidirectional, in that lower mood at the time of completing a PROM impacts on how an individual remembers their experience of their condition, which affects the scores [45]. Psychological health status also fluctuates over time, with research demonstrating a within-person fluctuation over short periods of time [35].

### Variation in scores

Variation in scores is dependent on several internal processes an individual uses to complete a measurement tool. Completion of an outcome measurement is reliant on the ability of individuals to appraise their condition which involves a cognitive process. The internal processes (integration) involved for each individual when appraising their condition is influenced by an individual’s cognitive process and their recall. As completion of a PROM requires individuals to reflect on their health, there is a degree of recall involved which impacts on and is impacted by how individuals integrate their experience. All of these concepts then lead to what is completed on the measurement tool and the interpretation of outcome scores.

Within-person variance was commonly observed for different mood disorders in daily and weekly scores, including suicidal ideation [42], eating disorders [55], bipolar and borderline personality disorder [27]. Cognitive decline and an increase in fatigue during the day is observed in MS patients affecting their performance to do tasks [30, 35], with substantial moment-to-moment and day-to-day fluctuations in fatigue severity found in relapse-remitting MS patients [41]. This decline in cognitive function can affect the internal processes involved in responding to an outcome measure, ultimately affecting the PROM score.

The sensitivity of the measurement to detect any changes in outcomes over time, and how change is defined to be clinically important within studies were important issues discussed in the articles [35, 39]. Diaries were more sensitive to daily score changes than measures obtained by patient interview, for pain intensity for cancer patients [48], and for young people with juvenile idiopathic arthritis [22]. The timing of measurements has been shown to be of significant importance, particularly with conditions that affect cognitive performance, such as MS patients demonstrating cognitive fatigue declining as the day progresses [30].

Daily measurements of mood, in one study, impacted the evaluation of health outcomes when measuring efficacy of psychopharmacological or psychological interventions [27]. However, daily measurements can also affect how individuals report their symptoms, for example in one study, pain significantly decreased during the second week of the study, which may have been an unintentional feedback intervention resulting in changes in their appraisals or pain management [26]. Although we do not know if the changes were also due to the fact that pain naturally decreased, thus representing true variation in outcomes.

Recall bias is when patients remember an event or experience incorrectly [57]. Retrospective accounts can lead to misclassification of symptoms [20], and an over-estimation of symptoms [37, 48]. Psychological health status [40], symptoms at the time of recall [23], length of the recall period, and primacy or recency of information [37] all impact on how individuals appraise their condition. A systematic review of studies on major depressive disorders revealed that negative recall bias in these patients exist mostly in the under-reporting of negative affect [34]. Asking patients to summarise their mood over a requested period potentially overlooks clinically meaningful differences in symptom patterns which could be picked up at each moment in time [37]. Although pain scores were higher in the evening and fluctuated across the weeks, pain recall was inaccurate for cancer patients with over-estimation of pain reported from a previous week [48].

## Discussion

This scoping review provides evidence for cyclical variation of PROMs for certain conditions, mainly respiratory, musculoskeletal, mental health and nervous system. The literature demonstrates a range of periodic fluctuations (e.g. diurnal, circadian, infradian and seasonal) across these conditions. Key concepts important in explaining cyclical variation of PROMs were extracted from the literature, and a conceptual model developed. The conceptual model provides a foundation in explaining the factors affecting variation in PROMs scores.

The concepts within the model were categories under four main aspects: core constructs, mediator, moderator and determinants. The model identifies the core constructs as variation in health outcomes (PROs), and variation in scores (PROMs), a key mediator (psychological health status), determinants impacting on a core construct (disease-related biorhythms, timing of biomedical interventions) and moderators (individual and environmental factors). Variation in outcomes and scores was found to be mediated by individual/environmental factors, and psychological health status at the time of completing a PROM.

All the included studies used quantitative methods to collect momentary and retrospective accounts of patient experience. In order to collect momentary accounts of individuals’ experiences authors used an ecological momentary assessment approach to data collection. However, many of the authors raised issues in relation to recall bias when questionnaire items required participants to provide a retrospective account of their health, consisting with the cognitive literature [58, 59]. According to cognitive science, our experiences, albeit good and bad, are encoded as an overall evaluation capturing the remembered intensity of the experience [58, 60] and memory is influenced by the individual’s context and mental state at the time of recall.

Alongside the periods of time PROMs require patients to reflect on, the frequency by which researchers or clinicians measure health may be important to consider especially with regard to day-to-day fluctuations [45]. Individual patients also exhibit different fluctuations, with individually-specific triggers and understanding this could explain these patterns in chronic conditions. This would help both patients and clinicians to efficiently manage the progression of diseases. However, there was a lack of patient perspective corroborating the hypothesised concepts within the model, due to the study designs identified.

Repeated measurements and a qualitative examination into the effect of time would provide better insight into the everyday correlates of patients’ symptoms and the contributing factors to fluctuations in outcome scores, such as quality of sleep or other symptoms (e.g. mood) [41].

Another factor to consider is how time was handled during the analysis of the data and what type of statistical tests were performed to analyse the data. Appropriate methodological approaches to analysing the data are necessary when modelling the effects of time. Research within the chronobiological field recommends to first plot the data as a function of time and use statistical techniques (i.e. spectral analysis) for detecting periodic patterns in time-related data [61, 62]. Cornelissen [61] highlights that classical study designs encouraging fewer test groups (or testing points) are not powerful enough to detect a time effect in comparison to chronobiology studies where they recommend using at least six timepoints per cycle.

### Limitations

The studies included in the review used a diverse range of methodologies, albeit all quantitative. Some of the authors were not fully transparent on the methods or analyses used, which proved challenging when appraising the quality of the studies. A limitation of this review is the exclusion of articles that were not published in English. Another potential limitation is the use of the terms in the search strategy and whether the list was comprehensive or sensitive enough to capture all studies of interest (e.g. qualitative). Whilst developing the layout of the concepts in the model, a degree of subjectivity was needed, although this was an iterative exercise which was not done in isolation and the concepts were directly drawn from the articles.

### Implications

There are various factors to consider for clinicians and researchers when using PROMs to assess effectiveness of interventions and/or progression of a disease. As there are thousands of PROMs instruments available for use [63, 64], the type of measurement that is used should be sensitive enough to detect changes in scores for patients. Changes in scores, as demonstrated by the conceptual model, is dependent on the time (time of day or year) when a patient completes the measurement. The type of measurement and where these are taken may also impact on how patients complete them, for example before a doctor’s appointment in a healthcare setting or at home. Understanding the biorhythms of each condition and how that may affect physiological as well as self-reported data needs to be considered when interpreting results.

In addition, the frequency by which patient-reported outcome data is collected may present a more enhanced picture of the longitudinal impact of the condition. Kleiman et al. [29] stipulate that no single data point should be used in making clinical decisions as, for example, variations occur in suicidal ideation over the course of a few hours. As seen in de Wit’s [48] study daily diaries of pain experience showed variation of pain experience occurring on a daily basis. Multiple measurements can provide clinicians with a better understanding as to when patients are most vulnerable. This could enable clinicians to gauge when interventions would be most effective, providing a preventative rather than reactive approach to healthcare delivery. In addition, with multiple measurements patients would feel more empowered to better manage their conditions. An in-depth systematic identification of qualitative literature is needed to corroborate our findings and conceptual model, ideally supplemented with primary qualitative research using an analytical framework approach.

Many conditions are often seen in isolation of other co-morbidities when patients visit specific specialists, despite evidence demonstrating interacting effects of each condition [65]. With the rise of multimorbidity around the world the way healthcare is delivered should take the implications of multiple conditions on health outcomes into account [66, 67]. In addition, medication timing and type of medication impacts on how patients experience their condition over time, and report on that experience. Chronotherapeutics is a growing field of research demonstrating that timing of medication can alter the course or progression of a condition, which in effect can alter outcome scores [68, 69].

## Conclusion

There is evidence of the impact of biological rhythms on PROMs scores, with potentially significant implications for clinical assessments in the care for people with chronic conditions. The proposed conceptual model can support further research in this area.

## Supplementary Information

**Additional file 1**. Search strategy.

**Additional file 2**. Adapted CASP questions for quality checking.

## Data Availability

Not applicable.

## References

[1] Smolensky MH, Lamberg L (2000). The body clock: guide to better health.

[2] Purcell HJ, Gibbs JS, Coats AJ, Fox KM (1992). Ambulatory blood pressure monitoring and circadian variation of cardiovascular disease; clinical and research applications. Int J Cardiol.

[3] Smolensky M (1996). Chronobiology and chronotherapeutics applications to cardiovascular medicine. Am J Hypertens.

[4] Patrick Guyatt GH, Acquadro CD, Higgins JP (2008). Patient-reported outcomes. Cochrane handbook for systematic reviews of interventions.

[5] Valderas JM, Alonso J (2008). Patient reported outcome measures: a model-based classification system for research and clinical practice. Qual Life Res.

[6] Wilson IB, Cleary PD (1995). Linking clinical variables with health-related quality of life: a conceptual model of patient outcomes. J Am Med Assoc.

[7] Valderas JM, Alonso J, Prieto L, Espallargues M, Castells X (2004). Content-based interpretation aids for health-related quality of life measures in clinical practice. An example for the visual function index (VF-14). Qual Life Res.

[8] Porter I, Goncalves-Bradley D, Ricci-Cabello I, Gibbons C, Gangannagaripalli J, Fitzpatrick R (2016). Framework and guidance for implementing patient-reported outcomes in clinical practice: evidence, challenges and opportunities. J Comp Eff Res.

[9] Black N. Patient reported outcome measures could help transform healthcare. BMJ (Online). 2013. 10.1136/bmj.f167 23358487

[10] National Collaborating Centre for Mental Health. The Improving Access to Psychological Therapies Manual. 2019.

[11] Peters MD, Godfrey CM, Khalil H, McInerney P, Parker D, Soares CB (2015). Guidance for conducting systematic scoping reviews. Int J Evid Based Health.

[12] Victoor A, Delnoij DM, Friele RD, Rademakers JJ (2012). Determinants of patient choice of healthcare providers: a scoping review. BMC Health Serv Res.

[13] Gonçalves Bradley Gibbons C, Ricci-Cabello I, Bobrovitz NJH, Gibbons EJ, Kotzeva A, Alonso J, Fitzpatrick R, Bower P, van der Wees PJ, Rajmil L, Roberts NW, Taylor RS, Greenhalgh J, Porter I, Valderas JMDC (2015). Routine provision of information on patient-reported outcome measures to healthcare providers and patients in clinical practice (Protocol). Cochrane Database Syst Rev.

[14] McHugh ML (2012). Interrater reliability: the kappa statistic. Biochem Medica.

[15] Ouzzani M, Hammady H, Fedorowicz Z, Elmagarmid A (2016). Rayyan: a web and mobile app for systematic reviews. Syst Rev.

[16] Hu X, Rousseau R, Chen J (2011). On the definition of forward and backward citation generations. J Informetr.

[17] World Health Organisation; International classification of diseases for mortality and morbidity statistics (11th Revision). 2018. Available from: https://icd.who.int/browse11/l-m/en

[18] Programme CAS. CASP Checklists - CASP - Critical Appraisal Skills Programme [Internet]. 2019 [cited 2019 Oct 3]. Available from: https://casp-uk.net/casp-tools-checklists/

[19] Valderas JM, Goncalves D, Alonso J (2012). A model for patient reported outcomes. Qual Life Res.

[20] Abdel-Kader K, Jhamb M, Mandich LA, Yabes J, Keene RM, Beach S (2014). Ecological momentary assessment of fatigue, sleepiness, and exhaustion in ESKD. BMC Nephrol.

[21] Dekkers JC, Geenen R, Godaert GL, van Doornen LJ, Bijlsma JW (2000). Diurnal rhythm of salivary cortisol levels in patients with recent-onset rheumatoid arthritis. Arthritis Rheum.

[22] Bromberg MH, Connelly M, Anthony KK, Gil KM, Schanberg LE (2016). Prospective mediation models of sleep, pain, and daily function in children with arthritis using ecological momentary assessment. Clin J Pain.

[23] Okifuji A, Bradshaw DH, Donaldson GW, Turk DC (2011). Sequential analyses of daily symptoms in women with fibromyalgia syndrome. J Pain.

[24] Hamilton NA, Catley D, Karlson C (2007). Sleep and the affective response to stress and pain. Health Psychol.

[25] Feuerecker R, Habs M, Dieterich M, Strupp M (2015). Chronic subjective dizziness: fewer symptoms in the early morning: a comparison with bilateral vestibulopathy and downbeat nystagmus syndrome. J Vestib Res.

[26] Stinson JN, Stevens BJ, Feldman BM, Streiner D, McGrath PJ, Dupuis A (2008). Construct validity of a multidimensional electronic pain diary for adolescents with arthritis. Pain.

[27] Tsanas A, Saunders KE, Bilderbeck AC, Palmius N, Osipov M, Clifford GD (2016). Daily longitudinal self-monitoring of mood variability in bipolar disorder and borderline personality disorder. J Affect Disord.

[28] Houtveen JH, Lipovsky MM, Kool M, Sorbi M, Buhring ME, van Broeckhuysen-Kloth S (2015). The day-to-day concurrence of bodily complaints and affect in patients with severe somatoform disorder. Scand J Psychol.

[29] Kleiman EM, Turner BJ, Fedor S, Beale EE, Huffman JC, Nock MK (2017). Examination of real-time fluctuations in suicidal ideation and its risk factors: results from two ecological momentary assessment studies. J Abnorm Psychol.

[30] Claros-Salinas D, Bratzke D, Greitemann G, Nickisch N, Ochs L, Schroter H (2010). Fatigue-related diurnal variations of cognitive performance in multiple sclerosis and stroke patients. J Neurol Sci.

[31] Curran SL, Beacham AO, Andrykowski MA (2004). Ecological momentary assessment of fatigue following breast cancer treatment. J Behav Med.

[32] Partridge MR, Karlsson N, Small IR (2009). Patient insight into the impact of chronic obstructive pulmonary disease in the morning: an internet survey. Curr Med Res Opin.

[33] McCarley C, Hanneman SK, Padhye N, Smolensky MH (2007). A pilot home study of temporal variations of symptoms in chronic obstructive lung disease. Biol Res Nurs.

[34] Aan Het Rot M, Hogenelst K, Schoevers RA (2012). Mood disorders in everyday life: a systematic review of experience sampling and ecological momentary assessment studies. Clin Psychol Rev.

[35] Feys P, Gijbels D, Romberg A, Santoyo C, Gebara B, de Noordhout BM (2012). Effect of time of day on walking capacity and self-reported fatigue in persons with multiple sclerosis: a multi-center trial. Multi Scler.

[36] Kratz AL, Ehde DM, Bombardier CH, Kalpakjian CZ, Hanks RA (2017). Pain acceptance decouples the momentary associations between pain, pain interference, and physical activity in the daily lives of people with chronic pain and spinal cord injury. J Pain.

[37] Pfaltz MC, Michael T, Grossman P, Margraf J, Wilhelm FH (2010). Instability of physical anxiety symptoms in daily life of patients with panic disorder and patients with posttraumatic stress disorder. J Anxiety Disord.

[38] Sewell L, Singh SJ, Williams JE, Morgan MD (2010). Seasonal variations affect physical activity and pulmonary rehabilitation outcomes. J Cardiopulm Rehabil Prev.

[39] Vernon M, Kline Leidy N, Nacson A, Nelsen L (2010). Measuring cough severity: development and pilot testing of a new seven-item cough severity patient-reported outcome measure. Ther Adv Respir Dis.

[40] Lavender JM, De Young KP, Wonderlich SA, Crosby RD, Engel SG, Mitchell JE (2013). Daily patterns of anxiety in anorexia nervosa: associations with eating disorder behaviors in the natural environment. J Abnorm Psychol.

[41] Powell DJH, Liossi C, Schlotz W, Moss-Morris R (2017). Tracking daily fatigue fluctuations in multiple sclerosis: ecological momentary assessment provides unique insights. J Behav Med.

[42] Graham-Engeland JE, Zawadzki MJ, Slavish DC, Smyth JM (2016). Depressive symptoms and momentary mood predict momentary pain among rheumatoid arthritis patients. Ann Behav Med.

[43] Shin JH, Lee G (2014). Seasonal changes in symptoms in patients with chronic prostatitis/chronic pelvic pain syndrome: a seasonal follow-up study. Scand J Urol.

[44] Crosby RD, Wonderlich SA, Engel SG, Simonich H, Smyth J, Mitchell JE (2009). Daily mood patterns and bulimic behaviors in the natural environment. Behav Res Ther.

[45] Schanberg LE, Gil KM, Anthony KK, Yow E, Rochon J (2005). Pain, stiffness, and fatigue in juvenile polyarticular arthritis: contemporaneous stressful events and mood as predictors. Arthritis Rheum.

[46] Schlager D, Froom J, Jaffe A (1995). Winter depression and functional impairment among ambulatory primary care patients. Compr Psychiatry.

[47] Schwartz AL (2000). Daily fatigue patterns and effect of exercise in women with breast cancer. Cancer Pract.

[48] de Wit R, van Dam F, Hanneman M, Zandbelt L, van Buuren A, van der Heijden K (1999). Evaluation of the use of a pain diary in chronic cancer pain patients at home. Pain.

[49] Hardt J, Gerbershagen HU (1999). No changes in mood with the seasons: observations in 3000 chronic pain patients. Acta Psychiatr Scand.

[50] Roche N, Chavannes NH, Miravitlles M (2013). COPD symptoms in the morning: impact, evaluation and management. Respir Res.

[51] Kikuchi H, Yoshiuchi K, Yamamoto Y, Komaki G, Akabayashi A (2012). Diurnal variation of tension-type headache intensity and exacerbation: an investigation using computerized ecological momentary assessment. Biopsychosoc Med.

[52] Bellamy N, Sothern RB, Campbell J, Buchanan WW (1991). Circadian rhythm in pain, stiffness, and manual dexterity in rheumatoid arthritis: relation between discomfort and disability. Ann Rheum Dis.

[53] Lavender JM, De Young KP, Anestis MD, Wonderlich SA, Crosby RD, Engel SG (2013). Associations between retrospective versus ecological momentary assessment measures of emotion and eating disorder symptoms in anorexia nervosa. J Psychiatr Res.

[54] Schwartz. Daily Fatigue Patterns and Effect of Exercise in Women with Breast Cancer. 1065. 10.1046/j.1523-5394.2000.81003.x 10732535

[55] Bromberg MH, Connelly M, Anthony KK, Gil KM, Schanberg LE (2014). Self-reported pain and disease symptoms persist in juvenile idiopathic arthritis despite treatment advances: an electronic diary study. Arthritis Rheumatol.

[56] Dekkers JC, Geenen R, Godaert GLR, Doornen LJP, Bijlsma JWJ (2000). Diurnal courses of cortisol, pain, fatigue, negative mood, and stiffness in patients with recently diagnosed rheumatoid arthritis. Int J Behav Med.

[57] Althubaiti A (2016). Information bias in health research: definition, pitfalls, and adjustment methods. J Multidiscip Healthc.

[58] Bryant RA (1993). Memory for pain and affect in chronic pain patients. Pain.

[59] Streiner Norman GRDL (2008). Health measurement scales—a practical guide to their development and use.

[60] Ariely D (1998). Combining experiences over time: the effects of duration, intensity changes and on-line measurements on retrospective pain evaluations. J Behav Decis Mak.

[61] Cornelissen G (2014). Cosinor-based rhythmometry. Theor Biol Med Model.

[62] Stroebel AM, Bergner M, Reulbach U, Biermann T, Groemer TW, Klein I (2010). Statistical methods for detecting and comparing periodic data and their application to the nycthemeral rhythm of bodily harm: a population based study. J Circadian Rhythm.

[63] Mapi Research Trust. PROQOLID. 2002. Available from: https://eprovide.mapi-trust.org/

[64] IMIM Foundation. BiblioPRO International. 2021. Available from: https://www.biblioprointernational.org/en/

[65] Porter I, Davey AF, Gangannagaripalli J, Evans J, Bramwell C, Gibbons C, et al. Integrating Patient Reported Outcome Measures (PROMs) into routine nurse-led primary care for patients with multimorbidity: a feasibility and acceptability study. Heal Qual Life Outcomes. 2021;in press. 10.1186/s12955-021-01748-2 PMC8074460 33902607

[66] Valderas JM, Gangannagaripalli J, Nolte E, Boyd CM, Roland M, Sarria-Santamera A (2019). Quality of care assessment for people with multimorbidity. J Intern Med.

[67] Valderas JM, Mercer SW, Fortin M (2011). Research on patients with multiple health conditions: different constructs, different views, one voice. J Comorbidity.

[68] Smolensky MH, Reinberg AE, Martin RJ, Haus E (1999). Clinical chronobiology and chronotherapeutics with applications to asthma. Chronobiol Int.

[69] Elliott WJ (2001). Timing treatment to the rhythm of disease. A short course in chronotherapeutics. Postgr Med.

