# Supplementary material for: Development of a conceptual model for research on cyclical variation of patient reported outcome measurements (PROMs) in patients with chronic conditions: a scoping review
Source: J Patient Rep Outcomes. 2021 Nov 4;5:117. doi: 10.1186/s41687-021-00395-x (PMC8568745; doi:10.1186/s41687-021-00395-x)
Supplement: Supplementary file 1 — Additional file 1. Search strategy. [file 41687_2021_395_MOESM1_ESM.docx]

Supplementary material 1: Search strategy

| **Domain** | **Medline ^** | **EMBASE ^** | **PsycINFO ^** | **CINAHL ^^** |
| --- | --- | --- | --- | --- |
| **Patient reported outcomes (PROs)** | 1 health status.tw. | 1 health status.tw. | 1 health status.tw. | S1 TI health status or AB health status |
|  | 2 quality of life.tw. or exp quality of life/ or quality of life.mp. | 2 quality of life.tw. or exp quality of life/ or quality of life.mp. | 2 quality of life.tw. or exp quality of life/ or quality of life.mp. | S2 TI quality of life or AB quality of life or MH quality of life |
|  | 3 (QL or QoL or HRQL or HRQoL).tw. | 3 (QL or QoL or HRQL or HRQoL).tw. | 3 (QL or QoL or HRQL or HRQoL).tw. | S3 TI (QoL or HRQL or HRQoL) or AB (QoL or HRQL or HRQoL) |
|  | 4 patient-reported.tw. | 4 patient-reported.tw. | 4 patient-reported.tw. | S4 TI patient reported or AB patient reported |
|  | 5 (function* adj2 (status or psychological or mental or physical or social)).tw. | 5 (function* adj2 (status or psychological or mental or physical or social)).tw. | 5 (function* adj2 (status or psychological or mental or physical or social)).tw. | S5 TI (function* N2 (status or psychological or mental or physical or social)) or AB (function* N2 (status or psychological or mental or physical or social)) |
|  | 6 disabilit*.tw. | 6 disabilit*.tw. | 6 disabilit*.tw. | S6 TI disabilit* or AB disability* |
|  | 7 activities of daily living.tw. | 7 activities of daily living.tw. | 7 activities of daily living.tw. | S7 TI activities of daily living or AB activities of daily living |
|  | 8 (wellbeing or well being).tw. | 8 (wellbeing or well being).tw. | 8 (wellbeing or well being).tw. | S8 TI (wellbeing or well being) or AB (wellbeing or well being) |
|  | 9 (happi* or happy).tw. | 9 (happi* or happy).tw. | 9 (happi* or happy).tw. | S9 TI (happi* or happy) or AB (happi* or happy) |
|  | 10 pain.tw. | 10 pain.tw. | 10 pain.tw. | S10 TI pain or AB pain |
|  | 11 fatigue.tw. | 11 fatigue.tw. | 11 fatigue.tw. | S11 TI fatigue or AB fatigue |
|  | 12 (shortness adj2 breath).tw. | 12 (shortness adj2 breath).tw. | 12 (shortness adj2 breath).tw. | S12 TI (shortness N2 breath) or AB (shortness N2 breath) |
|  | 13 dyspn?ea.tw. | 13 dyspn?ea.tw. | 13 dyspn?ea.tw. | S13 TI dyspn?ea) or AB dyspn?ea |
|  | 14 cough.tw. | 14 cough.tw. | 14 cough.tw. | S14 TI cough or AB cough |
|  | 15 dizz*.tw. | 15 dizz*.tw. | 15 dizz*.tw. | S15 TI dizz*or AB dizz* |
|  | 16 insomnia.tw. | 16 insomnia.tw. | 16 insomnia.tw. | S16 TI insomnia or AB insomnia |
|  | 17 anorexi*.tw. | 17 anorexi*.tw. | 17 anorexi*.tw. | S17 TI anorexi* or AB anorexi* |
|  | 18 nausea.tw. | 18 nausea.tw. | 18 nausea.tw. | S18 TI nausea or AB nausea |
|  | 19 cognitive function.tw. or exp cognitive function/ or cognitive function.mp. | 19 cognitive function.tw. or exp cognitive function/ or cognitive function.mp. | 19 cognitive function.tw. or exp cognitive function/ or cognitive function.mp. | S19 TI cognitive function or AB cognitive function or MH cognitive function |
|  | 20 (cognitive adj2 performance*).tw. | 20 (cognitive adj2 performance*).tw. | 20 (cognitive adj2 performance*).tw. | S20 cognitive n2 performance or AB cognitive n2 performance |
|  | 21 (neurobehavio* adj2 performance*).tw | 21 (neurobehavio* adj2 performance*).tw | 21 (neurobehavio* adj2 performance*).tw | S21 neurobehavio* n2 performance* or AB neurobehavio* n2 performance* |
|  | 22 (symptom? adj2 (assessment or index or indices or instrument? or measure? or profile? or rating? or report* or scale? or schedule? or scor* or survey?)).tw. | 22 (symptom? adj2 (assessment or index or indices or instrument? or measure? or profile? or rating? or report* or scale? or schedule? or scor* or survey?)).tw. | 22 (symptom? adj2 (assessment or index or indices or instrument? or measure? or profile? or rating? or report* or scale? or schedule? or scor* or survey?)).tw. | S22 TI (symptom? n2 (assessment or index or indices or instrument? or measure? or profile? or rating? or report* or scale? or schedule? or scor* or survey?)) or AB (symptom? n2 (assessment or index or indices or instrument? or measure? or profile? or rating? or report* or scale? or schedule? or scor* or survey?)) |
|  | 23 1 or 2 or 3 or 4 or 5 or 6 or 7 or 8 or 9 or 10 or 11 or 12 or 13 or 14 or 15 or 16 or 17 or 18 or 19 or 20 or 21 or 22 | 23 1 or 2 or 3 or 4 or 5 or 6 or 7 or 8 or 9 or 10 or 11 or 12 or 13 or 14 or 15 or 16 or 17 or 18 or 19 or 20 or 21 or 22 | 23 1 or 2 or 3 or 4 or 5 or 6 or 7 or 8 or 9 or 10 or 11 or 12 or 13 or 14 or 15 or 16 or 17 or 18 or 19 or 20 or 21 or 22 | S23 S1 or S2 or S3 or S4 or S5 or S6 or S7 or S8 or S9 or S10 or S11 or S12 or S13 or S14 or S15 or S16 or S17 or S18 or S19 or S20 or S21 or S22 |
|  |  |  |  |  |
| **Measurement** | 24 (index or indices).tw. | 24 (index or indices).tw. | 24 (index or indices).tw. | S24 TI (index or indices) or AB (index or indices) |
|  | 25 profile.tw. | 25 profile.tw. | 25 profile.tw. | S25 TI profile or AB profile |
|  | 26 rating.tw. | 26 rating.tw. | 26 rating.tw. | S26 TI rating or AB rating |
|  | 27 scale.tw. | 27 scale.tw. | 27 scale.tw. | S27 TI scale or AB scale |
|  | 28 schedule.tw. | 28 schedule.tw. | 28 schedule.tw. | S28 TI schedule or AB schedule |
|  | 29 survey.tw. | 29 survey.tw. | 29 survey.tw. | S29 TI survey or AB survey |
|  | 30 questionnaire*.tw. | 30 questionnaire*.tw. | 30 questionnaire*.tw. | S30 TI questionnaire* or AB questionnaire* |
|  | 31 health surveys.mp. | 31 health surveys.mp. | 31 health surveys.mp. | S31 TX health survey |
|  | 32 24 or 25 or 26 or 27 or 28 or 29 or 30 or 31 | 32 24 or 25 or 26 or 27 or 28 or 29 or 30 or 31 | 32 24 or 25 or 26 or 27 or 28 or 29 or 30 or 31 | S32 S24 or S25 or S26 or S27 or S28 or S29 or S30 or S31 |
|  |  |  |  |  |
| **Time** | 33 (biolog* adj2 clock*).tw. | 33 (biolog* adj2 clock*).tw. | 33 (biolog* adj2 clock*).tw. | S33 TI biolog* n2 clock* or AB biolog* n2 clock* |
|  | 34 periodicity.tw. | 34 periodicity.tw. | 34 periodicity.tw. | S34 TI periodicity or Ab periodicity |
|  | 35 chronobiolog*.tw. | 35 chronobiolog*.tw. | 35 chronobiolog*.tw. | S35 TI chronobiolog* or AB chronobiology* |
|  | 36 time-of-day.tw. | 36 time-of-day.tw. | 36 time-of-day.tw. | S36 TI time-of-day or AB time-of-day |
|  | 37 chronotype*.tw. | 37 chronotype*.tw. | 37 chronotype*.tw. | S37 TI chronotype* or AB chronotype* |
|  | 38 circadian.tw. | 38 circadian.tw. | 38 circadian.tw. | S38 TI circadian or AB circadian |
|  | 39 (sleep-wake adj2 cycle*).tw. | 39 (sleep-wake adj2 cycle*).tw. | 39 (sleep-wake adj2 cycle*).tw. | S39 TI sleep-wake n2 cycle or AB sleep-wake n2 cycle |
|  | 40 twenty-four hour rhythm*.tw. | 40 twenty-four hour rhythm*.tw. | 40 twenty-four hour rhythm*.tw. | S40 TI twenty-four hour rhythm* or AB twenty-four hour rhythm* |
|  | 41 24-hour rhythm*.tw. | 41 24-hour rhythm*.tw. | 41 24-hour rhythm*.tw. | S41 TI 24-hour rhythm* or AB 24-rhythm* |
|  | 42 diurnal.tw. | 42 diurnal.tw. | 42 diurnal.tw. | S42 TI diurnal or AB diurnal |
|  | 43 (light dark adj2 cycle*).tw. | 43 (light dark adj2 cycle*).tw. | 43 (light dark adj2 cycle*).tw. | S43 TI light dark n2 cycle* or AB light dark n2 cycle* |
|  | 44 infradian.tw. | 44 infradian.tw. | 44 infradian.tw. | S44 TI infradian or AB infradian |
|  | 45 (tidal adj2 rhythm*).tw. | 45 (tidal adj2 rhythm*).tw. | 45 (tidal adj2 rhythm*).tw. | S45 TI seasonal* or AB seasonal* |
|  | 46 seasonal*.tw. | 46 seasonal*.tw. | 46 seasonal*.tw. | S46 TI (morning* or TI evening*) or AB (morning* or TI evening*) |
|  | 47 (morning* or evening*).tw. | 47 (morning* or evening*).tw. | 47 (morning* or evening*).tw. | S47 TI (awakening or waking) or AB (awakening or waking) |
|  | 48 (awakening or waking).tw. | 48 (awakening or waking).tw. | 48 (awakening or waking).tw. | S48 TI (nighttime or night-time) or AB (nighttime or night-time) |
|  | 49 (nighttime or night-time).tw. | 49 (nighttime or night-time).tw. | 49 (nighttime or night-time).tw. | S49 TI nocturnal or AB nocturnal |
|  | 50 nocturnal.tw. | 50 nocturnal.tw. | 50 nocturnal.tw. | S50 TI ultradian or AB ultradian |
|  | 51 ultradian.tw. | 51 ultradian.tw. | 51 ultradian.tw. | S51 TI time course or AB time course |
|  | 52 time course.tw. | 52 time course.tw. | 52 time course.tw. | S52 TI diary or AB diary |
|  | 53 diary.tw. | 53 diary.tw. | 53 diary.tw. | S53 TI experience sampling method* or AB experience sampling method* |
|  | 54 experience sampling method*.tw. | 54 experience sampling method*.tw. | 54 experience sampling method*.tw. | S54 TI ecological n2 momentary n2 assessment* or AB ecological n2 momentary n2 assessment* |
|  | 55 (ecological adj2 momentary adj2 assessment*).tw. | 55 (ecological adj2 momentary adj2 assessment*).tw. | 55 (ecological adj2 momentary adj2 assessment*).tw. |  |
|  | 56 33 or 34 or 35 or 36 or 37 or 38 or 39 or 40 or 41 or 42 or 43 or 44 or 45 or 46 or 47 or 48 or 49 or 50 or 51 or 52 or 53 or 54 or 55 | 56 33 or 34 or 35 or 36 or 37 or 38 or 39 or 40 or 41 or 42 or 43 or 44 or 45 or 46 or 47 or 48 or 49 or 50 or 51 or 52 or 53 or 54 or 55 | 56 33 or 34 or 35 or 36 or 37 or 38 or 39 or 40 or 41 or 42 or 43 or 44 or 45 or 46 or 47 or 48 or 49 or 50 or 51 or 52 or 53 or 54 or 55 | S55 S33 or S44 or S35 or S36 or S37 or S38 or S39 or S40 or S41 or S42 or S43 or S44 or S45 or S46 or S47 or S48 or S49 or S50 or S51 or S52 or S53 or S54 |
|  |  |  |  |  |
| **Chronic conditions** | 57 (chronic adj2 (illness* or condition* or disease*)).tw. | 57 (chronic adj2 (illness* or condition* or disease*)).tw. | 57 (chronic adj2 (illness* or condition* or disease*)).tw. | S56 MH chronic disease |
|  | 58 (long-term adj2 (illness* or condition* or disease*)).tw. | 58 (long-term adj2 (illness* or condition* or disease*)).tw. | 58 (long-term adj2 (illness* or condition* or disease*)).tw. | S57 TI (chronic disease or chronic illness or chronic conditions) or AB (chronic disease or chronic illness or chronic conditions) |
|  | 59 exp chronic disease/ | 59 exp chronic disease/ | 59 exp chronic disease/ | 58 TI (long term disease or long term condition or long term illness) or AB (long term disease or long term condition or long term illness) |
|  | 60 57 or 58 or 59 | 60 57 or 58 or 59 | 60 57 or 58 or 59 | S59 S56 or S57 or S58 |
|  |  |  |  |  |
| **PROS AND MEASUREMENT AND TIME AND CHRONIC CONDITIONS** | 61 23 and 32 and 56 and 60 | 61 23 and 32 and 56 and 60 | 61 23 and 32 and 56 and 60 | S60 S23 and S32 and S55 and S59 |
| ^ Accessed through OvidSP on 27.04.17; ^^ Accessed through EBSCO on 01.05.17; Truncation & wildcards for OvidSP based searches: * free text; ? one or none character; ? any character (for different spelling); adjn finds the words if they are within n words of one another regardless of the order in which they appear; Truncation & wildcards for CINAHL: * free text; ? any character (for different spelling); Nn finds the words if they are within n words of one another regardless of the order in which they appear. | | | | |
