# Supplementary material for: Development of a conceptual model for research on cyclical variation of patient reported outcome measurements (PROMs) in patients with chronic conditions: a scoping review
Source: J Patient Rep Outcomes. 2021 Nov 4;5:117. doi: 10.1186/s41687-021-00395-x (PMC8568745; doi:10.1186/s41687-021-00395-x)
Supplement: Supplementary file 2 — Additional file 2. Adapted CASP questions for quality checking. [file 41687_2021_395_MOESM2_ESM.docx]

Supplementary material 2: Adapted CASP questions for quality checking

| **Criteria** | **Scoring** |
| --- | --- |
| Was there an explicit hypothesis in relation to cyclical variation? | Yes (1)  Unclear (0)  No (-1) |
| Were the recruited participants representative of the general population? |  |
| Was the outcome a validated measurement? |  |
| Are we confident that participants completed the measurements as scheduled in the data collection procedures? |  |
| Were the data available for the whole period that is relevant to the proposed rhythm (e.g. 24-hr, 7 days, weekly)? |  |
| Have the confounding factors related to cyclical variation in PROMs been taken into account in the design/analysis? |  |
| Is attrition less than 20%? |  |
|  |  |
